# Species Identification of Rehabilitated Critically Endangered Orangutans Through DNA Forensic: Implication for Conservation

**DOI:** 10.21315/tlsr2024.35.1.7

**Published:** 2024-03-30

**Authors:** Christy Lavenia, Dwi Sendi Priyono, Donan Satria Yudha, Tuty Arisuryanti

**Affiliations:** 1 Department of Biology, Universitas Indonesia, Depok 16424. West Java, Indonesia; 2 Department of Tropical Biology, Faculty of Biology, Universitas Gadjah Mada. Jl. Teknika Selatan, Sinduadi. Mlati, Sleman, 55281. Special Region of Yogyakarta, Indonesia; 3 Centre for Indonesia Tropical Biodiversity (CENTROBIO), Faculty of Biology, Universitas Gadjah Mada. Jl. Teknika Selatan, Sinduadi. Mlati, Sleman, 55281. Special Region of Yogyakarta, Indonesia

**Keywords:** Conservation, Molecular Markers, Orangutan, Rehabilitation, Wildlife DNA Forensic

## Abstract

Rehabilitating and releasing orangutans back into the wild is one of the conservation strategies being pursued to conserve orangutans. However, the species determination between Sumatran, Tapanuli, and Bornean orangutans is essential for reintroduction to avoid outbreeding depression, which could lead to DNA hybridisation and increase the probability of recessive characters. Here, we reported on an investigation of three orangutans in which DNA forensic techniques were used to identify the species before release and reintroduction to their habitat. By applying DNA forensic, the orangutan was successfully confirmed with high probabilities (100%) by identifying two orangutan species, *Pongo abelii* and *Pongo pygmaeus wurmbii*. Based on ambiguous morphology, we found the possibility of orangutan species being misidentified in rehabilitation. This case report demonstrates the importance of molecular diagnostics to identify the orangutan species. We also provide workflow recommendations from genetic aspect for rehabilitated orangutans. These recommendations will enable decision-makers to consider genetics when assessing future management decisions, which will help ensure that the orangutan species is effectively conserved.

**Highlights**

- We found the possibility of orangutan species being misidentified in rehabilitation.
- This work demonstrates the benefits of utilising the ND5 marker for molecular diagnostics to identify the orangutan species.
- This paper provides recommendations from genetic aspects, including identification of geographic origin, species/sub-species/hybrid identification, genetic diversity, level of inbreeding, and kinship analysis, to support more effective and appropriate orangutan rehabilitation programs.

## INTRODUCTION

Numerous conservation initiatives aim to improve the size of the orangutan population, which is critically endangered. The rehabilitation of orangutans is one of the conservation projects applied to orangutans. Rehabilitation is the strategy where the captive animals are “handled for physical and physiological disabilities until they recover health, are managed to help them possess natural social and ecological skills, and are able to wean themselves from human contact and dependence, such that they can survive independently (or with greater independence) in the wild” (Beck *et al*. 2007). Reintroduction is an initiative to improve a species in a region that was once part of its historic range but from which it has disappeared. After some rehabilitation procedures, orangutans can be released into the wild.

Almost all wild-born orangutans who get rehabilitation were illegally seized as infants by killing their mothers (Peters 1995). They originate from every region of Borneo and Sumatra that is currently inhabited. The next post-rehabilitation problem is identifying the species of orangutans to reintroduce into the correct location (Russon 2009). Initially, the orangutan has differentiated taxonomically into two different subspecies, *Pongo pygmaeus abelii* and *Pongo pygmaeus pygmaeus* (Jalil *et al*. 2008; Goossens *et al*. 2009). However, recent data indicated that there are three different species of orangutans, namely the Bornean orangutan (*P. pygmaeus*), Sumatran orangutan (*P. abelii*), and Tapanuli orangutan (*P. tapanuliensis*) based on morphometric, behavioral, and genomic analysis (Nater *et al*. 2017).

Species determination is essential to maintain the genetic integrity of the species. The mistaken species determination can result in outbreeding depression, in which the hybrid is not well adapted to either environment or can decline reproductive fitness (Frankham *et al*. 2002). Outbreeding depression could lead to DNA hybridisation, which increases the probability of recessive characters not being adaptive (Zhang *et al*. 2001). Unfortunately, in some cases, the morphology of orangutans appears confusing (Palmer *et al*. 2021), resulting in potential for species misidentification.

Conservation and biodiversity management have greatly benefited from species identification using molecular taxonomy (Maldonado *et al*. 2015; Priyono *et al*. 2018; 2020; 2023). This study uses the mitochondrial gene ND5, which codes for the NADH-ubiquinone oxidoreductase chain 5 protein to identify species. The mtDNA control region sequence was used long ago to analyze primates’ population diversity (Zhang *et al*. 2001). This research aims to identify a protocol for rehabilitating orangutans to assist with assigning orangutans the correct species identification. We also investigated how this genetic aspect could contribute to forensic case investigations in rehabilitated orangutans.

## MATERIALS AND METHODS

**Figure 1:**
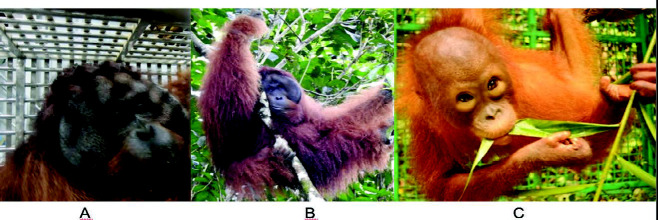
Three rehabilitated orangutans were used in this genetic analysis for species identification. (a) Sample 1; (b) sample 2 ppr; and (c) sample 3. (Source: East Kalimantan Agency for Conservation of Natural Resources 2021)

Staff from the East Kalimantan Agency for Conservation of Natural Resources under the Directorate of Conservation of Natural Resources and Ecosystems of the Ministry of Environment and Forestry through the Balikpapan Agricultural Quarantine Center sent three orangutan blood samples stored in EDTA vacutainer tubes (letter no. 2021.1.1800.K13.K.002010) from Java, which is not their natural habitat. Identifying these three orangutans (Fig. 1) aims to confirm the species and geographic origin for reintroduction purposes. DNeasy Blood and Tissue extraction kit was used on blood samples following standard procedure by the manufacturer (Qiagen 2020). EDTA blood samples were stored at −20°C until it is ready to be used. Each sample’s DNA concentration was measured using NanoDrop 2000 (Nanodrop Technologies; Wilmington, DE, USA).

Polymerase chain reaction (PCR) reaction cocktail contains 2x QIAGEN Multiplex PCR Master Mix, 0.15x Q-Solution, 1.5 mmol MgCl_2_, and 10 mM primers. PCR was conducted for 35 cycles at 94°C for 40 s, 56°C for 40 s, and 72°C for 30 s, with 94°C 12 min before and 72°C 10 min after that cycle. Applying ND5-specific primers, the ND5 region of mitochondrial DNA was amplified (forward) ND5r and 5’-TAA-CCG-CCC-TCA-CCT-TAA-CTT-CCC-3’ (reverse) 5’-GGT-CAG-GAT-GAA-GCC-AAT-GTC-G-3’ (Zhang *et al*. 2001). An approximately 550 bp fragment ND5 near the 5’-region was amplified using the primers. The PCR product was then purified using the QIAquick PCR Purification Kit (Qiagen, Germany) before the sequencing process.

Purified products were sequenced on both DNA strands at 1st Base, Singapore. In addition to ND5 data, orangutan DNA sequences from other mitochondrial regions have been previously reported. The Geneious programme (Kearse *et al*. 2012) was used to edit sequences and assemble contigs. Sequence alignment was verified by BLASTn (https://blast.ncbi.nlm.nih.gov/). The presence of fixed molecular markers among orangutan species was established by inspection of aligned mtDNA sequences. jModelTest v.2.1.7 (Darriba *et al*. 2012) determined which DNA substitution model best fit our data set using Akaike’s Information Criterion (AIC; Sakamoto *et al*. 1986). The Hasegawa-Kishino-Yano (HKY) + gamma (G) model was selected out of 88 candidate models with a log-likelihood of ln = 825.262 (α = 0.031). The Bayesian Inference (BI) method was used to construct phylogenetic trees. Estimated parameters were implemented to the Bayesian analysis conducted with Metropolis-coupled Markov chain Monte Carlo algorithms (MCMC: 10^7^) as implemented in BEAST (Bouckaert *et al*. 2014). The iTOL version 3 (Letunic & Bork 2016) was used to visualise the Bayesian phylogenetic tree.

## RESULTS

The DNA sequence obtained using the sequencing analysis is 550 bp long. To confirm that nuclear mitochondrial DNA segments (NUMTs) was not present in the obtained DNA sequences, we carefully verified them manually. No internal stop codons or gaps were detected on the sequence obtained, and Ti/TV ratios were 18.2. Aligned Sequences (all 427 base pairs long) matched nucleotide positions in the *Pongo abelii* isolate PAB-Sinjo mitochondrion partial genome with GenBank accession number: KU353726.1 from sites 11890 to 12316. We successfully identified three rehabilitated orangutan samples. The result of sequence DNA indicated that three rehabilitated orangutans were *P. abelii* (samples 1 and 2) and *P. pymnaeus wurmbii* (sample 3) with accession number KU353726.1, MN186981.1, and AF255451.1. The sequences in this study were confirmed 100% by the GenBank database with high percentage of identity (Table 1). In addition, the ND5 marker revealed significant genetic distances across different species of orangutans (Table 2). The *P. abelii* and *P. tapanuliensis* orangutans have the highest genetic distance (0.072). In contrast, the *P. pygmaeus wurmbii* and *P. pygmaeus morio* orangutans, which still belong to the same species, have the lowest genetic distance (0.005).

**Figure 2:**
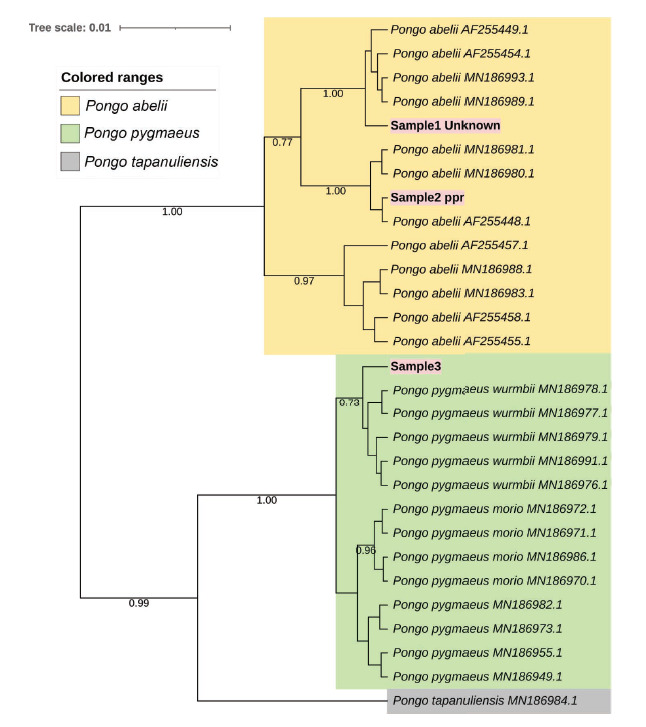
Bayesian phylogenetic tree based on ND5 region of mitochondrial DNA. Hence, the statistical support is reported at each node (posterior probability).

There are 43 DNA polymorphic sequences in the sample and the GenBank database sequences. We also found 23 diagnostic nucleotides from the total sequence unique to each orangutan species (Table 3). Further, the phylogenetic tree formed also shows monophyletic grouping based on orangutan species. The phylogenetic trees showed that, with a high posterior probability, the query sequences clustered strongly with the known sequences of *P. abelii* and *P. pygmaeus wurmbii* (1.00) (Fig. 2). Interestingly, we detected the possibility of morphological misidentification; sample 2 ppr was previously assumed to be *P. pygmaeus wurmbii*. However, phylogenetic and BLASTn analysis consistently showed that the individual was *P. abelii*.

## DISCUSSION

Wildlife forensic techniques are the scientific approaches and techniques used to investigate crimes involving wildlife, such as poaching, illegal hunting, the trade in endangered species, and wildlife trafficking. These approaches are used to gather and examine several types of evidence, including animal remains, DNA samples, and other trace materials, to determine the species concerned, determine the cause of death, and obtain data that can be utilised in court cases (Cooper & Cooper 2013; Walker & Adrian 2014). In aspects of the identification process in wildlife forensics, the taxonomic species involved in the crime, as well as its conservation and CITES status, are primarily considered. Only after completing this process would it be feasible to determine the actual consequences of the crime on a populational or even landscape scale, to punish the alleged perpetrators accountable for their acts, and to take effective action (Bourret *et al*. 2020; Gouda *et al*. 2020; Meiklejohn *et al*. 2021). Regardless of the circumstance, there are two main approaches to performing a proper species determination: morphological and molecular.

Developing analysis tools that can provide DNA evidence to aid in conservation law enforcement is one area of conservation genetics that has long been acknowledged but is now garnering more attention. In this case, identification aims to determine the species precisely before being released back into their natural habitat. Field officers could not identify samples 1 and 3 because of confusing morphological characters. Wild-born orangutans in rehabilitation do not have their geographic origins defined, which has complicated comparisons of three species of orangutans, *Pongo pygmaeus*, *Pongo abeli* and *Pongo tapanuliensis*. Identification based on morphological description can be so subjective to differing interpretations. The uncertainties and validity of orangutan species identification are still debated (Ryder & Chemnick 1993). According to previous studies (MacKinnon 1974; Warren *et al*. 2001), some general external features can be used to distinguish between the two orangutan species. Red to deep maroon or blackish brown hair has been described as having been found on Bornean orangutans. Sumatran orangutans, in comparison, have lighter hair that is rusty red or light cinnamon. However, be cautious when using colour as a taxonomic diagnostic in orangutans. There is an extreme type of Sumatran orangutan with dark hair that mimics Bornean orangutans (Courtenay 1988). In the present study, the molecular identification successfully confirmed three orangutans with high statistical confidence.

In this investigation, samples 1 and 3 were previously difficult for the field team to identify, but sample 2 ppr was determined to be *P. pygmaeus wurmbii*. However, using this molecular marker, we could identify and validate the species that the identification of the sample 2 ppr had proven to be. Genetics can be an objective species identification and guide for the management development in a rehabilitation center or captivity (Kamaluddin et al. 2018). In addition to ND5 data, orangutan DNA sequences from other mitochondrial regions were identified at San Diego Zoo (Zhang *et al*. 2001). Previously, (Perwitasari-Farajallah 2009) showed the effectiveness of using ND5 as a rapid protocol to discriminate against two species of orangutans, *P. pgymaeus* and *P. abelii* for rehabilitation centers and zoos. Field staff can update and revise the collection records according to clear clade separation utilising ND5 marker, high genetic distance, and robust Bayesian inference support.

Some DNA markers have also demonstrated the ability to distinguish between different orangutan subspecies. One of the markers used for identification is the D-loop which has succeeded in identifying the subspecies level of captive orangutans in Peninsular Malaysia (Kamaluddin *et al*. 2018). Although the orangutan captive studbooks may be unclear in some cases and have hybrid potential (Cocks 2007; Gippoliti & D’Alessandro 2013; Palmer *et al*. 2021; Banes *et al*. 2022), this marker can be a good option for the orangutan subspecies identification because it has relatively shorter base length of 305 bp. Another molecular marker is the von Willebrand factor (vWF) gene (Abdul-Manan *et al*. 2020). This marker can differentiate orangutan species but is still unclear for subspecies identification. In addition, nuclear DNA markers are somewhat challenging to amplify for specimens with degraded or limited DNA conditions so that mitochondrial DNA can offer more significant advantages for forensic geneticists (Melton *et al*. 2012; Syndercombe Court 2021).

**Figure 3:**
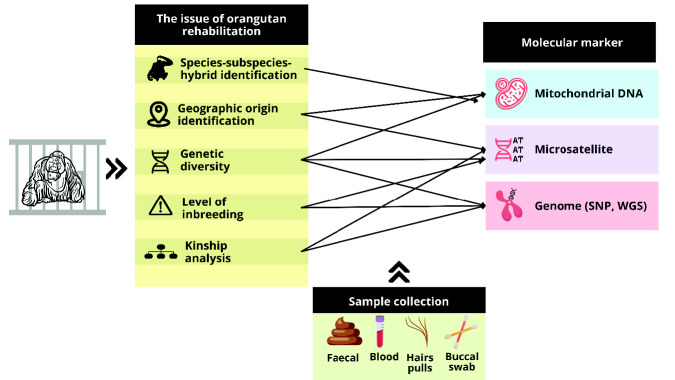
Guideline recommendation from the genetic aspect for conservation management in rehabilitated orangutans.

Orangutans have been collected and confiscated from many areas. The information and accurate genetic and morphological tests must determine the orangutan species. Genetic testing could determine the provenance to the species level of rehabilitated animals (Russon 2009). Based on circumstances the genetic aspect can access, we develop a workflow of recommendations for conservation management in orangutans under rehabilitation (Fig. 3). We divide some important genetic aspects:

1. **Geographic origin identification**. Determining the population or region from whence a person was taken should make it easier to spot illegal trade routes and harvesting activities, which would help prioritise efforts to prevent or end poaching (Manel *et al*. 2002). In addition, when an animal’s geographic origin is unknown, it may be challenging to identify the population that would be a suitable recipient of their release. Specific local adaptations may be beneficial for native animals (Tallmon *et al*. 2004), while rehabilitated, individuals from different localities may not have these physical or physiological features that would enhance their chances of surviving or successfully reproducing at the relocation site. DNA has aided conservation management in some rehabilitated primate species to determine the geographic origin, e.g., in chimpanzees (Goldberg 1997) and howler monkeys (Oklander *et al*. 2020). Therefore, it is crucial to carefully relocate or release rehabilitated orangutans into their natural habitat with the aid of DNA methods.
2. **Species/subspecies/hybrid identification**. Some issues complicate the understanding of the morphological variations and classification of orangutans. It is essential to avoid breeding different species together in captivity if the offspring of those orangutans may be released into an area where orangutans coexist. Therefore, it is critical to determine what species already inhabit areas before introducing orangutans there. Several molecular markers that can be used to identify orangutan subspecies include: D-loop (Kamaluddin *et al*. 2018), and vWF gene (Abdul-Manan *et al*. 2020). It implies that differentiating between orangutan species is essential for a clear breeding strategy and preserving purebred populations. This is so that breeders can maintain the natural intraspecific variation found in feral populations without introducing foreign traits that cannot be eliminated afterward once subspecific hybrids are produced. Unfortunately, due to its maternal inheritance, mitochondrial DNA is a poor diagnostic for identifying hybrid individuals (Hurst & Jiggins 2005). Diagnostic markers that can be used for hybrid detection that have been carried out for primates are karyotyping (Nieves *et al*. 2008), genomic (Hirai *et al*. 2017), a combination of microsatellite and Y-chromosome markers (Cortés-Ortiz *et al*. 2007; 2010). Therefore, it is essential to identify the species/ subspecies/ hybrid of orangutans kept in each facility before they are included in any conservation or reintroduction effort.
3. **Genetic diversity**. By increasing the size and genetic diversity of existing populations or establishing new, self-sustaining populations in suitable environments, translocations strive to maintain the long-term survival of threatened species (Weeks *et al*. 2011). However, a single population with lower genetic diversity is especially vulnerable and has high disease-related mortality rates (e.g., Bertola *et al*. 2022). Regarding management, it would be advisable to relocate individuals based on information about the genetic diversity of orangutans that have undergone rehabilitation. Microsatellites and mitochondrial DNA in howler monkeys are two examples of molecular markers that can be utilised to assess genetic diversity in primates (Goossens *et al*. 2002; Wang *et al*. 2015). This strategic approach would conserve the orangutan’s genetic background and act as a preventative measure to contain a potential disease in the future.
4. **Level of inbreeding**. An important aspect that affects the successful reintroduction of species under rehabilitation is inbreeding. Concern may arise from the level of inbreeding when a few rehabilitated orangutans interbreed within the facilities of rehabilitation centres. Inbred animals may be less fit and possess deleterious alleles in homozygous form. When the proper confiscation records are unavailable, the situation could become even more precarious. In this situation, it might be challenging to distinguish inbred offspring from wild-caught individuals. The level of inbreeding can be assessed using STR and microsatellite markers (Widdig *et al*. 2017; Oklander *et al*. 2021).
5. **Kinship analysis**: In situations where lineage can be more accurately documented, such as in captives (*ex-situ*), intensively managed wild (*in situ*), or semi-wild populations of animals, pedigrees have proven to be very useful for managing the genetic structure of these populations. Kinship analysis has been completed using microsatellite and SNP markers (Warren *et al*. 2000; Banes *et al*. 2020). The optimal method for managing gene flow involves using mean kinship data within and between populations (derived via modeling, pedigrees, genetic markers, or genomes), and transferring individuals among fragments with the lowest mean kinships between populations. Following that, populations should be observed to ensure that gene flow has reached the appropriate levels, and that genetic diversity has increased.

The collection of DNA material from orangutans is an essential endeavor that enables researchers to gain valuable insights into the genetic diversity and evolutionary history of orangutans. Many sources of DNA material, such as blood, hair, buccal swabs, and fecal material, can be used for genetic testing on orangutans (Da Silva *et al*. 2012; Zemanova 2019). In this investigation, we also discovered a nucleotide diagnostic that can provide useful information for conservation management. It is possible to identify individuals’ populations of origin with certainty using nucleotide diagnostics that show unique nucleotide substitutions in one population but not in others (Yang *et al*. 2007; Wong *et al*. 2009; McTavish & Hillis 2015). Matrilineal ancestry in great apes and monkeys has been investigated thoroughly due to the application of nucleotide diagnostics in mitochondrial DNA (Kopp *et al*. 2015; Rianti *et al*. 2015)

Genetic methods have been proven to help conservation management in rehabilitation of animals. It is necessary to establish standard guidelines to support conservation action plan strategies. The importance of releasing orangutans into natural habitats based on genetics has also been emphasised in the Strategy Action Plan for orangutan conservation in Indonesia for 2007–2017 to ensure prevention of genetic pollution. Developing a database system will be crucial for forensic cases, translocation or release programmes, pedigree information, and particularly for orangutan genetics. However, until now, there have been no standardised guidelines for managing orangutans by forensic DNA, either in Indonesia or Malaysia, so it needs to be developed jointly with academics, researchers, and policymakers. Specificity, sensitivity, integrity maintenance, recovery efficiency, impact on analytical assays, and methodologies should all be considered while validating protocols. An integrated strategy for law enforcement is necessary in accordance with the most recent CITES discussions on this species, and we end by urging range of countries to cooperate to give improved wildlife forensic law enforcement tools to eliminate this illegal trade.

## CONCLUSION

The popularity of wildlife DNA forensics emphasizes the worrying extent to which illegal activity is threatening endangered species and the expanding accessibility of DNA analysis. In this study, mitochondrial DNA could be used for rapid species discrimination of orangutans. This protocol could be easily applied to rehabilitation centres and zoos to resolve species determination problems. We note the potential for forensic DNA established in other primates that could be used in orangutan rehabilitation, including features of geographic origin, species identification, genetic diversity, level of inbreeding, and kinship analysis. Finally, standardised guidelines for orangutan rehabilitation need to be established, as we found there is a potential for species misidentification.

## Figures and Tables

**Table 1:** Sample verification on GenBank database using BLASTn.

Sample code	Putative	BLAST result	% identity	Description (no. access)
Sample 1	unknown	*P. abelii*	100	*P. abelii* isolate PAB-Sinjo (KU353726.1)
Sample 2 ppr	*P. pymnaeus wurmbii*	*P. abelii*	100	*P. abelii* isolate OU26(MN186981.1)
Sample 3	unknown	*P. pygmaeus pygmaeus*	100	*P. pygmaeus pygmaeus bor1* (AF255451.1)

**Table 2:** Genetic distance of orangutan species using ND5 marker.

Species	*P. abelii*	*P. pygmaeus morio*	*P. pygmaeus wurmbii*	*P. tapanuliensis*
*P. abelii*	-	-	-	-
*P. pygmaeus morio*	0.063	-	-	-
*P. pygmaeus wurmbii*	0.062	0.005	-	-
*P. tapanuliensis*	0.072	0.044	0.044	-

**Table 3:** Unique nucleotides are found in each orangutan species based on the ND5 marker.

Species	Diagnostic nucleotide	

	Site	Nucleotide
*P. abelii*	16	C
	121	A
	181	A
	258	T
	358	G
	376	C
	403	G
	424	T
*P. pygmaeus*	100	T
	73	G
	185	G
	190	C
	196	T
	268	C
*P. tapanuliensis*	146	T
	166	T
	172	T
	217	A
	227	C
	232	T
	322	T
	394	T
	409	A
